# A Multibranch of Convolutional Neural Network Models for Electroencephalogram-Based Motor Imagery Classification

**DOI:** 10.3390/bios12010022

**Published:** 2022-01-03

**Authors:** Ghadir Ali Altuwaijri, Ghulam Muhammad

**Affiliations:** 1 Department of Computer Engineering, College of Computer and Information Sciences (CCIS), King Saud University, Riyadh 11543, Saudi Arabia; 438203980@student.ksu.edu.sa; 2 Computer Sciences and Information Technology College, Majmaah University, Al Majma’ah 11952, Saudi Arabia; 3 Centre of Smart Robotics Research (CS2R), King Saud University, Riyadh 11543, Saudi Arabia

**Keywords:** electroencephalography (EEG), motor imagery (MI), deep learning (DL), Convolutional Neural Networks (CNN), brain computer interfaces (BCI)

## Abstract

Automatic high-level feature extraction has become a possibility with the advancement of deep learning, and it has been used to optimize efficiency. Recently, classification methods for Convolutional Neural Network (CNN)-based electroencephalography (EEG) motor imagery have been proposed, and have achieved reasonably high classification accuracy. These approaches, however, use the CNN single convolution scale, whereas the best convolution scale varies from subject to subject. This limits the precision of classification. This paper proposes multibranch CNN models to address this issue by effectively extracting the spatial and temporal features from raw EEG data, where the branches correspond to different filter kernel sizes. The proposed method’s promising performance is demonstrated by experimental results on two public datasets, the BCI Competition IV 2a dataset and the High Gamma Dataset (HGD). The results of the technique show a 9.61% improvement in the classification accuracy of multibranch EEGNet (MBEEGNet) from the fixed one-branch EEGNet model, and 2.95% from the variable EEGNet model. In addition, the multibranch ShallowConvNet (MBShallowConvNet) improved the accuracy of a single-scale network by 6.84%. The proposed models outperformed other state-of-the-art EEG motor imagery classification methods.

## 1. Introduction

With the introduction of sophisticated machine learning algorithms, high-performance computers, edge and cloud computing, and next-generation communication technologies, smart healthcare has become a reality [1,2]. Today, the biggest gap between humans and machines is being bridged with the use of brain–computer interfaces (BCIs). Advances in this field enable computers to be deliberately managed by brain signal activity monitoring [3]. Because it is non-invasive, has a fast time resolution, allows for user versatility, and is relatively inexpensive, an electroencephalogram (EEG) sensor is widely used in BCI systems to track brain signals [4]. Because EEG signals are nonstationary, they pose processing challenges; they are more likely to have signal artifacts, and they often suffer from external noise. EEG signals can also be influenced by a subject’s posture and mood. For example, an upright posture will, in general, improve concentration and EEG quality during recording. Also, when subjects are in an upright position, they have a stronger high-frequency content than when they are lying down or resting. It was additionally noted in [5] that postural changes in subjects who felt tired could increase their attention.

EEG signals are not the only signals used in brain research to monitor brain activity. Other signals used include magneto encephalogram (MEG) and magnetic resonance imaging (MRI). This paper focuses on EEG signals based on motor imagery (MI), which means imagining the movement of limbs without actually moving them. MI data are created when a subject imagines moving his or her right or left hand, or both hands, or his or her right or left foot, or both feet, or any of the five fingers, the tongue, or any limb in the body.

The electrode arrays used to record EEG signals are made up of a variety of sensor numbers, ranging from 3 to more than 500 electrodes. The number of electrodes or sensors used depends on the research aim. To make the experiment user-friendly, the researchers use an elastic cap, meshes, or rigid grids that the EEG electrodes are installed in. This type of configuration ensures that the data are collected from the same location on the scalp for each session in the experiment [6]. The reported data should be digitized and sent to an amplifier to be represented as a time series of voltage values. Amplification is necessary because EEG electrical signals have very low voltage.

An EEG signal that records the electrical activity of the brain from the scalp is a mixture of many underlying base frequencies. These frequencies reflect particular cognitive, emotional, or attentional states. The main frequencies of the human EEG waves (brain waves) are delta, theta, alpha, beta, and gamma, arranged from slowest to fastest brain waves. The speed of waves is measured in Hz (cycles per second). These brain waves change depending on what the subject is doing or feeling. When the subject feels slow, lazy, dreamy, or tired, the slower brain wave is dominant, whereas when the subject feels excited or hyper-alert, the faster brain wave is dominant. Although multiple brain waves may happen at the same time, only one brain wave will be dominant. Most research used the range between 0–35 Hz [7]. This paper focuses on using a raw EEG signal without any preprocessing, and uses the full band for the dataset.

EEG recording is a non-invasive and low-cost (compared with other related devices) method of recording. The first EEG device used on humans was invented in the 1920s by German neurologist Hans Berger [8]. It recorded electrical activity in the brain using electrode sensors. These electrodes were placed on the scalp surface in specific locations to record the electrical data.

**Figure 1:**
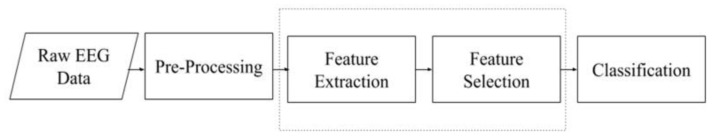
Typical MI EEG-based signal processing and classification system.

Researchers in the early 2000s found that the best way to classify EEG-based MI data involved using common spatial patterns (CSP). The idea of the CSP algorithm is to find a set of linear transformations, which are regarded as spatial filters, to maximize the distance in multiple classes. These classes are the motor imagery recorded during an MI-EEG task for the right hand, left hand, and feet. Following the estimation of the spatial filters, the relative energy of the filtered channels is calculated as the data representation. This high-dimensional EEG data representation can then be easily fed into a linear classifier such as a support vector machine (SVM), yielding high accuracy [9]. Figure 1 shows the main parts of the typical MI EEG-based signal processing and classification system.

The filter-bank CSP (FBCSP) is an extended technique of the CSP algorithm commonly used in MI-EEG decoding. It has won multiple EEG decoding competitions, including the BCI competition IV in both the 2a and 2b datasets [10]. Because not all frequency bands contain discriminatory information, the technique calculates the CSP energy characteristics for each of the temporally filtered signal outputs after passing it through a filter bank. After that, the features are chosen and classified. The additional step of conducting CSP on each filtered input enhances the classifier’s efficiency, and demonstrates the advantages of signal decomposition before calculating the spatial filter. The signal is reduced from a time series to a single value in that representation, so the temporal information is lost, and the dynamics of the signals, which may contain significant EEG information, are neglected.

As a growing area of interest in the field of BCI, the classification of MI-EEG is not only associated with significant potential, but also with important applications (e.g., robotics [11,12], therapeutic applications [13,14], and gaming [15]). Nevertheless, there are major limitations in terms of data collection and classification methods, and a particular challenge emerges from the problem of additional refinement required for systems undergoing clinical trials. This paper aims to devise an end-to-end classification model using deep learning that can classify MI-EEG-based signals with good accuracy and better kappa values. 

Despite deep learning (DL)’s growing success in many fields, its use in classifying EEG signal-based motor imagery has not yet yielded satisfactory results. It is challenging to create an ideal EEG classification model using DL because of the high dimensionality of EEG data (multichannel and sampling rate), noise, channel correlation, and artifact presence (i.e., motion). A data preparation stage must precede such a model framework, in which data must be extracted from the raw EEG signal, and translated into a new representation without significant loss of information. In turn, the deep learning network architecture must be built based on this representation to extract significant features from the input data. From this perspective, this paper explores suitable and efficient representations of raw EEG signals that can be fed into a DL-based EEG MI classification system.

Based on the preliminary observation, the main problem of EEG MI classification is that it is a subject-specific task. This means that each subject has different features that make the system more efficient in classifying the MI movement. Therefore, we propose in this paper a DL-based EEG MI classification model that can deal with the subject-specific task. The main contributions of the paper are as follows:

- Develop an end-to-end EEG MI classification model using deep learning that can deal with the subject-specific problem.
- Investigate which kernel size or filter size can extract good features for classification from all subjects.
- Fuse parallel models of EEGNet [16] and ShallowConvNet [16] to incorporate the influence of different filter sizes.
- Use multiple datasets to validate the proposed model.

The paper is organized as follows. We summarize the related research publications on MI-EEG classification algorithms, and MI datasets in Section 2. The proposed multibranch EEGNet and ShallowConvNet models (MBEEGNet, MBShallowConvNet) are presented in Section 3, and the experimental results are presented in Section 4 with a discussion. Finally, the paper is concluded in Section 5.

## 2. Background

### 2.1. Related Works

A single processing block can be utilized in deep learning to perform the whole feature extraction, selection, and classification pipeline. Convolutional Neural Networks (CNNs) [17,18,19,20,21] are the most commonly used architecture in MI EEG processing, but recurrent neural networks (RNNs) [18], stacked autoencoders (SAEs) [19], and deep belief networks (DBN) [18] have also been used.

The nature of MI EEG signals gives an advantage to CNN compared to other deep learning architectures. MI EEG signals are nonlinear and nonstationary. They have temporal and spatial features coming from the time spent imagining the movement and, at the same time, the record from different electrodes (each electrode has different locations that contain the spatial information). For this reason, CNNs have several advantages in MI EEG data processing: (i) raw data can be inputted into the system, thereby eliminating the need for prior feature extraction; (ii) the ability to learn temporal and spatial features at the same time; (iii) the ability to exploit the hierarchical nature of certain signals; (iv) high accuracy on large datasets.

Deep learning models are used in many fields today, one of which is EEG-based MI. Most of the papers that classify EEG-based motor imagery using deep learning can be divided into four approaches depending on the formulation of input. The input formulation can be extracted features, spectral images, raw signal values, or topological maps [7]. The architecture of the deep learning model played a big role in deciding which input formulation to use.

Tang et al. [20] compared the performance of handcrafted models and a deep CNN model on an EEG MI classification task. They built a CNN model consisting of five layers. The first layer was the input layer, followed by two hidden layers, one fully connected (FC) layer, and the output layer. The first three layers represent the feature extraction part, whereas the last two layers represent the classification part. The authors used the same training and test set in three models: power + SVM, CSP + SVM, and autoregression (AR) + SVM to compare their method with other handcrafted models. Their result showed that the CNN model yielded better accuracy than the others in a private dataset with two subjects. 

In [22], Dose et al. used an end-to-end model with CNN layers for feature extraction, and an FC layer as a classifier. This model consisted of two CNN layers: the first one is responsible for convolution in the time axis, and the second one provides convolution in the EEG channel axis. The output was then sent to the FC layer after being reduced in an average pooling layer. Finally, a SoftMax layer served as a classifier, with several neurons depending on the number of classes in the data. This model was applied to raw EEG signals from the PhysioNet EEG Motor Movement/MI Dataset. Moreover, the authors used a global classifier, as well as a subject-specific classifier. In both classifiers, the model achieved better results than other work done with the same dataset at that time. 

Some researchers have applied methods to prepare data before inputting them into a CNN. One such method was described in [23] by Sakhavi et al. The authors used the FBCSP in the EEG signals, after which they extracted temporal features as an envelope from each signal before feeding them to a channel-wise (CW) convolution with channel-to-channel mixing (C2CM). C2CM is a CNN that adds a channel mixing to the CW-CNN network, which is a neural network responsible for the convolution across time for all feature channels, using a common kernel shape. The authors applied their method in the BCI competition IV-2a dataset, yielding an average accuracy of 74.46% from nine subjects in the dataset. This is considered a better result than other methods using the same dataset during that time. Xu et al. [24] used a wavelet transform in time-frequency images to extract features in specific channels. The CNN’s input in the paper consisted of images done by the wavelet transform. The CNN was composed of two layers, and the experiment was undertaken using two databases: dataset III from BCI competition II, and dataset 2a from BCI competition IV. The highest accuracy in the study was 92.75% when selecting two channels, C3 and C4, in the first dataset. In comparison, the average of the best accuracy in nine subjects in the second dataset was 85.59%. 

However, as a 3D CNN shows improvement in the classification of image/video processing applications, it is also used in EEG-based MI, as shown in [21]. In [21], Zhao et al. presented a multi 3D CNN to preserve spatial features with temporal ones. They represented 3D EEG as a sequence of 2D arrays according to the location of the electrodes (if there is no electrode in that place in the array, they are padded with 0), and after that, they used EEG’s temporal information to extend the array to a 3D array. The multi 3D CNN consisted of three different sizes of the receptive field (RF) 3D CNNs, which are large, medium, and small receptive field networks: LRF, MRF, and SRF, respectively. Also, the authors used a cropped strategy to increase the number of training and testing examples. Their results showed that the multi 3D CNN achieved better accuracy than each of the three single networks. They compared their results to three of the best models using the same dataset, finding that the multi 3D CNN outperformed tangent space of submanifold (TSSM) + SVM and FBCSP in terms of average kappa value. Comparison with C2CM from [23] indicated that they were almost the same in terms of average kappa value, but the multi 3D CNN obtained a lower standard deviation in different subjects, which means it is more robust.

A new approach to EEG signal classification was presented in [25] by Amin et al., who used multilayer CNNs with two different feature fusion models: a multilayer perceptron (MLP) and autoencoders. The authors investigated different levels of CNNs to extract the most significant features, and then they fused them before classification to improve the accuracy of EEG-based MI. The CNN models contained different numbers of convolution-pooling blocks: CNN-1, CNN-2, CNN-3, and CNN-4. These were pretrained on the high gamma dataset (HGD) to avoid overfitting. Both models were tested in two classification methods: subject-specific and cross-subject classification. Also, two datasets were used for the test: dataset 2a from BCI competition IV (BCID) and HGD. The result in subject-specific classification showed that the MCNN model, which used MLP as its fusion method, yielded greater accuracy in both datasets than the other comparative methods. Additionally, the method of fusing CNNs and cross-encoding autoencoders, named the CCNN model, was associated with greater accuracy in both datasets in cross-subject classification results.

We did not find previous research on using a raw MI-EEG signal as input for the 2D CNN with multibranch. The idea of multiple branches is not new; it was used in [21] and [26]. The authors used a multibranch architecture with a 3D CNN; the input was a 3D EEG signal, and they also applied a 3D filter. In our proposed method, we use a 2D input formulation that is raw data, and we apply two 1D filters, one for time and the other for space; we believe this will reduce the computational complexity compared to using the 3D filters, and make the model more able to deal with the subject-specific problem. Researchers found in [27] that flattened networks, which use just one-dimensional filters to cover all three dimensions in 3D, perform quite well as standard convolutional networks, while using substantially less computation. For example, if we have 10 1D filters of size 8 × 1 with a step of 100 in the temporal direction, we have 10 × 8 × 1 × 1× 100 = 8000 multiplications for the temporal 1D filters; for the spatial direction (channel-wise), if there are 10 1D filters of size 1 × 22 with a step size of 21, there will be 10 × 1 × 22 × 1 × 21 = 4620 multiplications. Therefore, there will be 12,620 multiplications for both 1D filters, which are much less than one 3D filter multiplications (10 × 8 × 22 × 3 × 100 = 528,000). So, the 3D filter is difficult to implement in real-time applications, but the 1D filter has less complexity. 

A ConvNet [28], which employs convolutional layers to extract temporal and spatial information, was the first interesting technique that used raw EEG data. The FBCSP [10] is the source of inspiration for this design. A convolution with a kernel of size (1, Nt) is conducted, followed by another convolution with a kernel of size (C, 1), where Nt corresponds to the number of time samples, and C is the number of channels. The collected features are then classified using a softmax layer. Similar architectures for MI were introduced in [17]. ShallowConvNet is a shallow convnet made up of two convolutional layers followed by classification layers. DeepConvNet is a deep architecture with additional aggregating layers following the convolutional layer. ShallowConvNet outperforms the state-of-the-art FBCSP. EEGNet was presented by [16] as a compact version of previous approaches. It is based on depth-wise convolution and a separable convolution that allows the number of parameters to be reduced.

Other related architectures have been proposed: one is presented by M. Riyad et al. in [29]. The first part of that model is the same as EEGNet, with two convolutional layers that act as a temporal and spatial filter, whereas the second part has the inception block. This block contains two convolutions with different kernel sizes, a pointwise convolution, and an average pooling.

In [30], the authors used temporal convolutional networks (TCNs) with EEGnet to boost the performance accuracy. A standard causal convolution can only expand the size of its receptive field linearly as the network depth increases, which can be a significant drawback because a big receptive field size requires either an exceptionally deep network or one with a very large kernel size. In contrast, TCNs employ a sequence of dilated convolutions, which allows the network to expand its receptive field exponentially in size, proportional to network depth, using a strategy of exponentially increasing dilation factors, D. In addition, unlike other time series classification networks, such as recurrent neural networks (RNNs), TCNs do not experience exploding or vanishing gradient difficulties when trained on large input sequences because TCNs use connectors in the residual blocks.

All these architectures respond to the drawbacks of EEGNet, which is too shallow and compact, limiting network capacity, which, in most situations, results in overfitting. Because of a degradation problem with DeepConvNet, performance is still low, even with a deeper network. As a result, we recommend adopting a multibranch model that learns characteristics from multiple branches. 

The basic concept for a multibranch model is that the raw or prepared input feeds to different subnetworks with different characteristics. In [31], the authors proposed a CP-MixedNet architecture that used multiscale EEG extracted features from a series of convolution layers, each of which extracts EEG temporal information at various scales. In [32], the authors develop a parallel spatial–temporal representation of raw EEG signals that takes advantage of the self-attention process to generate different spatial-temporal features. They encode spatial dependencies between MI EEG channels using the spatial self-attention module in particular. This eliminates the artifacts produced by human channel selection by combining information across all channels with a weighted summation. Moreover, the temporal self-attention module converts global temporal information into features for each sample time step, allowing high-level temporal features of MI EEG signals to be extracted in the time domain. In [33], the authors divided the raw signal into three band-limited signals by filtering the signal to different band ranges. They changed the temporal convolutional filter size in each band range so they were actually having nine parallel branches (three for each filter band). This resulted in a huge number of parameters (≥405 K) for one filter band, which means more than 1215 K training parameters for the whole system. This situation limits the use of the system in many applications. In addition, there was no variation in the filter size, so the effect of different neighborhoods in channels were not incorporated in the system. 

Another complicated framework was proposed in [34], which uses a temporal-spectral-based squeeze-and-excitation feature fusion network (TS-SEFFNet). They divide their model into three parts: the first one is the deep-temporal convolution block (DT-Conv block), which uses convolutions to extract high-dimension temporal representations from raw EEG data in a cascade architecture. The multispectral convolution block (MS-Conv block) is then run in parallel to capture discriminative spectral features from corresponding clinical sub-bands using multilayer wavelet convolutions. The last suggested block was the squeeze-and-excitation feature fusion block (SE-Feature-Fusion block), which was used to fuse deep-temporal and multispectral features into comprehensive fused feature maps. This highlights channel-wise feature responses by creating interdependencies between different domain features. Compared to existing multibranch, multiscale, and parallel networks, our proposed models leverage the fundamental aspect of multibranch with kernel size variation, thus boosting classification accuracy with low complexity and a small number of parameters. Table 1 summarizes the related work.

### 2.2. BCI Competition IV-2a Dataset 

**Figure 2:**
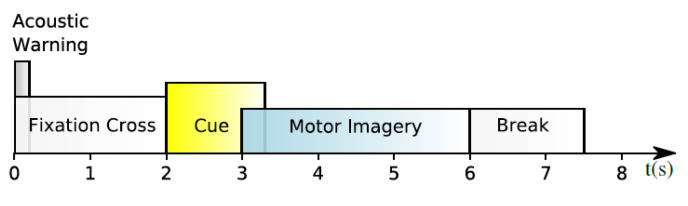
Timing pattern of the BCI Competition IV-2a.

It is one of the most used datasets in EEG-based MI classification. It is a publicly available dataset that was collected from nine subjects using 22 EEG electrodes at a sampling rate of 250 Hz. Then, it filtered using a bandpass filter between 0.5 and 100 Hz [35]. Furthermore, three extra electrooculography (EOG) channels were employed to collect data on eye movement. Four classes of imagined movements are contained in the dataset, each representing a different bodily part: left hand, right hand, feet, and tongue. Two sessions were recorded on different days from each subject. The first session was used for training purposes, whereas the second was used for testing purposes. Trials polluted with artifacts were excluded from each session’s 288 trials. All of the participants were seated in front of a computer screen on an armchair. A fixed cross appeared on a black screen at *t* = 0 for each trial, accompanied by a short warning tone to signify the start of a trial. A cue emerged in the shape of an arrow after two seconds (*t* = 2), pointing in the direction of one of the four classes: left means the left hand, right means the right hand, down means feet, and up means tongue. The arrow stayed in place for 1.25 s, prompting the subject to complete the desired class. When the fixed cross on the screen vanishes, this means that the subjects can relax and the trial is finished. Each trial was followed by a 2-s pause in which the screen was black. Figure 2 depicts the timing pattern for each trial.

### 2.3. High Gamma Dataset

With roughly 1040 trials (880 trials for training, and 160 for testing), the HGD comprises more trials than the BCI-IV 2a, with four classes (same as BCI-IV 2a): left and right hands, both feet, and rest. It was acquired from 14 volunteers (8 men, 6 women): 2 are left-handed, age 27.2 ± 3.6. The MI movement was executed by each subject at their own rate. In addition to the MI, according to the direction of a gray arrow on the screen, the subjects either clenched their toes repeatedly, tapped their fingers sequentially, or relaxed. These movements were chosen because they require minimum proximal muscle activation, yet are complicated enough to keep the patients active and engaged. The recordings were made in an EEG lab designed to collect data non-invasively using high-frequency, movement-related EEG components. A total of 128 channels were used to acquire the data, with a sampling frequency of 500 Hz. In each run, 80 arrows show, representing four classes. Each class included 260 trials, with a 4 s time window for each trial and 3–4 s inter-trial intervals [17].

## 3. Methodology

### 3.1. Data Preprocessing

The main components of a typical MI EEG-based classification system are preprocessing, feature extraction, and classification. The preprocessing step for raw EEG data removes noise and artifacts. Although it is applied in many systems, it is not a mandatory step. In contrast, feature extraction from EEG data is an important step before classification because it identifies which motor movement was imagined by the subject. This paper does not use significant preprocessing for raw data; we only extract the motor imagery signal time frame from the trial. No further bandpass filtering is used.

In this study, we used both the BCI Competition IV dataset 2a and the HGD. The first dataset used (BCI IV 2a) was recorded from 22 electrodes with a sampling frequency of 250 Hz. We extracted, from each trial, 0.5 s before the start of the pre-cue until the end of the trial; therefore, the total length we extracted from one trial is 4.5 s (250 × 4.5 = 1125 samples). Each channel was standardized, and no further prepossessing was used. Each trial was shaped like a dimensioned matrix (22,1125).

The second used dataset (HGD) was initially sampled at 500 Hz, before being resampled to 250 Hz. To remove unnecessary data, the number of channels was reduced from 128 to 44. The preprocessing stage was carried out according to the approach described in [36]. We took a length of 4.5 s from each trial, resulting in 1125 samples. The dimensions of the trial matrix were as follows (44,1125). Each channel was standardized, and no extra filters were used.

### 3.2. Proposed Models

The basic idea of the CNN is to analyze the influence of nearby neurons using a filter. The choice of filter size depends on the data type and feature map that we want to derive. The value for each point in the pattern is calculated based on the filter using a convolution operation. These feature maps are then taken through an activation function, which determines whether a certain feature is present at a given location.

CNNs, which formulate the convolution operation in the neural network context, address the problem of high-dimensional data, such as EEG signals [37]. The convolutional window is a small section of the input neurons to which each neuron in the first hidden layer of the CNN is connected. All neurons are given a bias, and each connection is given a weight. The window is then slid across the entire input sequence, and each neuron in the hidden layer learns to analyze a specific aspect of it. The kernel size is the size or length of the convolutional window. Instead of learning new weights and biases for each hidden layer neuron, the CNN now learns only one set of weights, and a single bias that is applied to all hidden layer neurons. This is the concept of weight sharing. We can describe this mathematically as:

(1) $$a_{ij}=f\left(b_{i}+\overset{k}{\underset{K=1}{\sum }}w_{iK}x_{j+K-1}\right)=f\left(b_{i}+W_{i}^{T}X_{j}\right)$$

where $a_{ij}$ is the activation or output of the *j*th neuron of the *i*th filter in the hidden layer, *f* corresponds to the activation function, *b_i_* is the shared overall bias of filter, *i*, *K* is the kernel size, *W_i_* = [*w_i_*_1_ *w_i_*_2_ … *w_ik_*] is a vector of the shared weights, and *X_j_* = [ *x_j_ x_j_*_+1_ … *x_j_*_+*k*−1_ ] is a vector of the output of the previse neurons, and T denotes the transpose operation. 

In motor imagery, the best kernel size differs from subject to subject, and for the same subject from time to time. To deal with the subject-specific issue in EEG MI classification using CNN, we proposed an EEG MI multibranch classification system; each branch has a different kernel size. This proposed method aims to find the appropriate convolution scale, that is, kernel size, for all subjects. Using different kernel sizes helps the method to be subject-specific, and makes the model more generalized.

There are three types of layers in a CNN: a convolutional layer (which gives the network its name), a pooling layer, and a fully connected layer. Each of these layers has various parameters that can be optimized, and that perform different tasks on the input data. The convolutional layer is used to extract features from input data. The convolution operation is a mathematical process that involves two inputs, such as an image matrix and a kernel or filter. It can preserve the relationship between pixels in an image. The same process is repeatedly applied to the input data with the same filter, resulting in a feature map or map of activations. This map indicates the locations and strength of a detected feature in the input data. The major building blocks in a CNN are the convolutional layers. 

The second layer is the pooling layer, which reduces the dimensionality of each map, but retains important information. There are several types of spatial pooling, which is also referred to as subsampling or downsampling. The most famous types are max pooling and average pooling. Finally, before the classification output of a CNN, the fully connected layers are used to flatten the outcomes before classification.

In any CNN, the most important parts are the number of layers, the activation function used, optimization algorithms, and dropout probability. The number of layers affects the kind and number of features that will be learned from the pattern, where each CNN layer learns filters of increasing complexity. Edges, corners, and other basic features are learned by filters in the first layers, whereas filters that detect parts of objects are learned by the middle layers. The general or higher representations are learned in the last layers.

Activation functions are used to determine and normalize the output. An activation function is a mathematical equation attached to each neuron in the network, which determines whether it should be activated or not. The activation of the neuron is based on whether the neuron’s input is relevant for the model’s prediction. Moreover, it normalizes the output to a range between −1 and 1, or between 0 and 1 of each neuron. There are three types of activation functions: binary step function, linear activation function, and nonlinear activation function. Real-world problems require nonlinear solutions to solve nontrivial problems. Nonlinear activation functions, which map input values to a desired range, are mostly used in deep learning networks to generate nonlinearity. Additionally, a deep neural network is required to learn complex datasets with high accuracy, which necessitates stacking multiple layers of neurons. This is a possibility when using nonlinear activation functions. There are several kinds of nonlinear activation functions, some of which can speed up the model. We use exponential linear units (ELUs) in our models.

Batch normalization is a technique of normalization that is performed between the layers of a neural network rather than on the raw data. Instead of normalizing the entire dataset, it is performed using mini-batches. Batch normalization helps accelerate training, makes learning easier, allows the use of higher learning rates, and regularizes the model [38]. Also, it helps to avoid overfitting. A cross-entropy optimizer, which is an algorithm or method for adjusting the properties of the neural network (e.g., weights and learning rate), is used to minimize the loss functions in our case. Those algorithms or techniques are responsible for minimizing losses, and producing the most accurate results possible. For the optimization algorithms in our models, we compare the stochastic gradient descent (SGD) and adaptive moment estimation (Adam), which are the most commonly used options in MI-EEG classification, and we find that Adam gives the best result in our case. Finally, the dropout probability is used to reduce the number of parameters by turning off some neurons.

The three important characteristics of the cerebral cortex, which are local connectivity, invariance to location, and invariance to local transition, can be mimicked using a CNN network. The literature revealed that the optimal kernel size varies from subject to subject, and from time to time (even for the same subject). Based on this, we proposed a multibranch CNN model. The model is built so that it can learn the temporal features from the first convolutional layer according to the temporal hierarchies of local and global modulations, whereas the spatial features can be learned in the second convolutional layer using the spatially global unmixing filters. For that, the input data are represented as a 2D array, where the rows represent the numbers of electrodes, and the columns are the number of time steps. The representation of the dataset of MI-EEG signals is:

(2) $$S={\left\{X_{i}\;,Y_{i}\right\}}_{i=1}^{n}$$

where *n* is the number of trails, *X_i_*, *Y_i_* are the signal and their corresponding class labels, and *Y_i_* ∈ {1, 2,*…*,*j*}, where *j* is the number of classes. *X* is represented as the input signal (it is a 2D array), *X* = [*E S*], where *E* refers to the number of EEG channels, and *S* to the length of EEG signal input. The output of the classification system is the output from the last layer, which is a softmax layer; it is a layer with a softmax activation function. The output from this layer is a vector with probabilities of each possible outcome or class. The sum of the probabilities for all possible outcomes or classes in the vector is one. We can define the softmax as:

(3) $$S{\left(v\right)}_{i}=\frac{e^{v_{i}}}{\sum_{j=1}^{n}e^{v_{i}}}$$

where *v* is the input vector to the softmax function, *S*; it contains n elements for *n* classes (outcomes), *v_i_* is the *i*th element in the input vector, *v*, and *n* is the number of classes.

The cost function or loss function is the categorical cross-entropy, which takes the output probabilities from the softmax function, and measures the distance from the true values; this gives a value of 0 or 1 for each class/outcome. Cross-entropy loss is used when adjusting model weights during training. The goal is to reduce the loss as much as possible; the smaller the loss, the better the model. The cross-entropy loss of a perfect model is zero. Other names for the cross-entropy loss function are logarithmic loss, log loss, or logistic loss. It is defined as:

(4) $$L_{CE}=-\overset{n}{\underset{i=1}{\sum }}t_{i}\log\left(p_{i}\right)$$

For *n* classes, where *t_i_* is the true label, *p_i_* is the softmax probability for the *i*th class, and the log is calculated to base 2.

**Figure 3:**
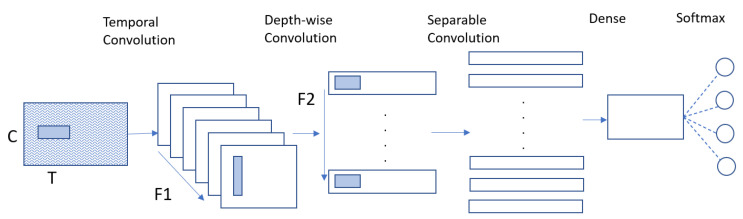
Architecture of the EEGNet model.

The proposed method can be divided into two parts: multibranch EEGNet (MBEEGNet) and multibranch ShallowNet (MBShallowNet). First, we implement the basic EEGNet and ShallowNet to find the three best kernel sizes for the first convolutional layer, and determine the optimal values for the hyperparameters. Those basic models contain blocks, the same as described in [16]. The EEGNet model learns frequency filters using a 2D temporal convolution, then utilizes a depth wise convolution to learn frequency-specific spatial filters. As demonstrated in Figure 3, separable convolution learns a temporal summary for each feature map separately before mixing the feature maps, and classifying them.

**Figure 4:**
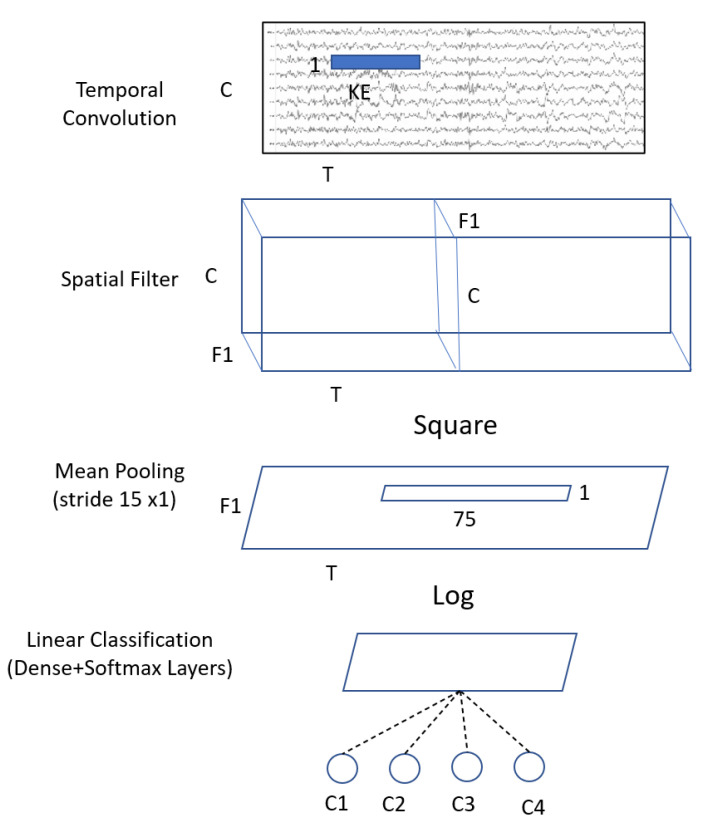
Architecture of the ShallowConvNet model where C = channels; T = time sample; KE = kernel size; F1 = number of filters; and C1,2,3,4 = classes.

Inspired by the FBCSP pipeline, the ShallowConvNet (Figure 4) is designed to decode band power features. The ShallowConvNet performs transformations that are comparable to the FBCSP operations. The first two layers perform a temporal convolution and a spatial filter, respectively. Next, a squaring nonlinearity, a mean pooling layer, and a logarithmic activation function were applied; these stages are equivalent to FBCSP’s trial log-variance computation. Unlike FBCSP, the ShallowConvNet encapsulates all computational processes in a single network, allowing all steps to be optimized simultaneously. This is the advantage of deep learning. In addition, because each trial has many pooling regions, the shallowConvNet can learn the temporal pattern of the band power fluctuations within the trial.

**Figure 5:**
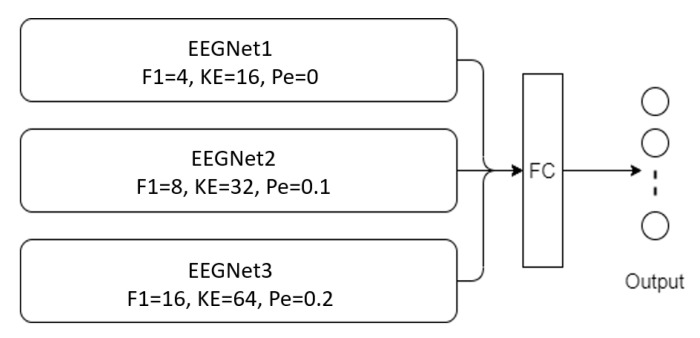
Architectural of MBEEGNet model (F1 = number of filters in temporal convolution, KE = kernel size in temporal convolution, Pe = dropout probability).

**Figure 6:**
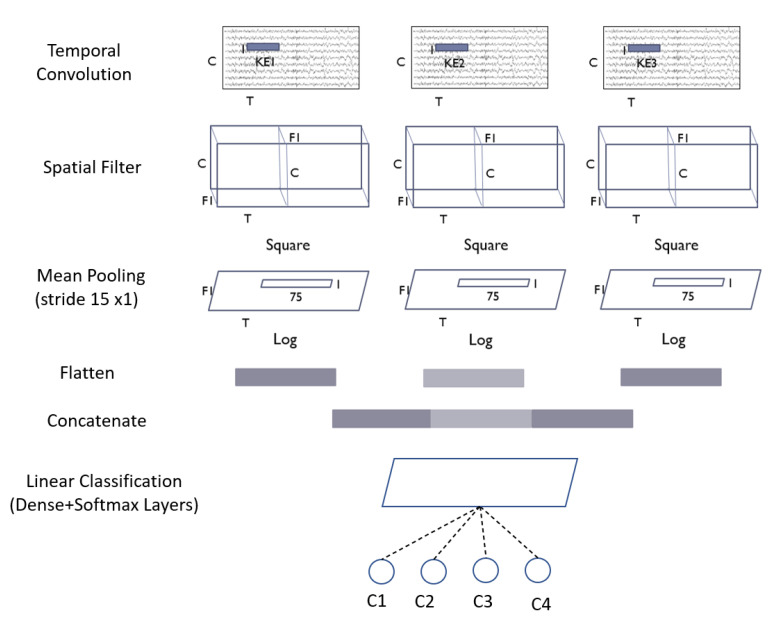
Architecture of MBShallowConvNet model.

After defining the three kernel sizes, and tuning the hyperparameters by implementing the basic models (EEGNet and ShallowConvNet), we follow the multibranch models. Those models take the raw EEG signal without any significant preprocessing as input, and contain three branches of deep learning networks, each with different kernel sizes. Then, we concatenate the output, and feed it to the softmax layer. The architecture of those models is shown in Figure 5 and Figure 6, respectively.

Each kernel size gives different information; from the combination of different kernel sizes, we can obtain information from all parts of the signal. We test our models in two different sets of benchmark datasets, BCI Competition IV-2a and HGD.

### 3.3. Training Procedure

The mental and physical health of subjects might vary greatly. There are two ways for classifying MI in EEG-MI research: within-subject and cross-subject. The within-subject strategy, which involves training and testing the model on sessions recorded for the same individual with different data, has recently achieved good accuracy [25]. The cross-subject strategy is used to train the model on all subjects before testing it on only one of them, and then repeating the process on the remaining subjects. However, due to the dynamic nature of each subject’s brain waves, the cross-subject approach remains problematic [25,36]. In this study, the proposed models are used to apply the within-subject method to the BCI-IV 2a dataset and the HGD dataset. One session is utilized for training, and the other is used for testing, in both datasets. Also, global parameters are employed for all subjects in the proposed models, as indicated in Table 2. During the training phase, a callback is utilized at the end of each epoch to save the best model weights based on the current best accuracy, and the best-saved model is loaded during the test phase. With a batch size of 64, and a learning rate of 0.0009, the model is trained for 1000 epochs. A cross-entropy error function and an Adam optimizer were used. We use the same training setting to train the models presented in this research.

## 4. Experimental Results

All experiments are implemented in Google’s Colab environment using the TensorFlow deep learning library with Keras API.

### 4.1. Performance Metrics

We used the following performance metrics to evaluate our models: *accuracy*, *precision*, *recall*, *F1 score*, and *Cohen’s kappa* test.

(5) $$Accuracy=\frac{TP+TN}{\left(TP+TN+FP+FN\right)}$$

(6) $$Precision=\frac{TP}{TP+FP},$$

(7) $$Recall=sensitivity=\frac{TP}{TP+FN}$$

Classification accuracy, the most used performance metric, is calculated using Equation (5). Then, the precision and the recall or sensitivity were obtained from Equations (6) and (7), respectively, where *TP* = true positive, *TN* = true negative, *FP* = false positive, and *FN* = false negative. The second most-used metric is Cohen’s kappa score, which is calculated using Equation (8).

(8) $$\mathrm{Cohen}’s\;\mathrm{kappa}=\frac{Po-Pe}{1-Pe}$$

where *Po* = total accuracy or the proportion of the observed agreement. *Pe* is calculated as Equation (9), where *C* = total number of the confusion matrix. We can also consider *Pe* as the probability that the agreement is due to chance.

(9) $$\mathrm{Pe}=\frac{\sum^{\;}\left(TP+FP\right)*\left(TP+FN\right)}{C^{2}}$$

For both the classification accuracy and the kappa scores, the standard deviation was calculated. The *F1* score is the final metric used, and it requires calculating both the precision and recall from Equations (6) and (7), respectively, before applying them to Equation (10).

(10) $$F1\;=\;2\;*\frac{Precision\;*\;Recall}{Precision+Recall}$$

### 4.2. Results of BCI Competition IV-2a Dataset

The proposed model was trained on session “T”, and tested on session “E” from the BCI Competition IV-2a data set. The subject-specific technique was utilized in the experiments.

Multiple metrics were used to compare the proposed model against state-of-the-art MI-EEG classification approaches, including classification accuracy, Cohen’s score, precision, recall, F1-score, and the number of parameters.

Table 3 presents the classification accuracy and kappa scores of each subject for several state-of-the-art MI-EEG algorithms employing a subject-specific approach on the BCI Competition IV-2a dataset. The suggested MBEEGNET and MBShallowConvNet, EEG-TCNet [30], both fixed and variable EEGNet [16,30], ShallowConvNet [16], and Incep-EEGNet [29] are the approaches compared. MBEEGNET and MBShallowConvNet, the proposed models, have an accuracy of 82.01% and 81.15%, respectively. The accuracy obtained by MBEEGNet was at least 9.61% higher than the accuracy obtained by the fixed model. When compared to the variable EEGNet model, which had an accuracy of 79.06%, the proposed model (MBEEGNet) had an improvement of 2.95%, even though the model employed fixed parameters for all individuals. The suggested model improved the accuracies of all the nine subjects in the EEGNet variable network, demonstrating that it is as good as or better than a variable network, while still employing fixed hyperparameters.

**Figure 7:**
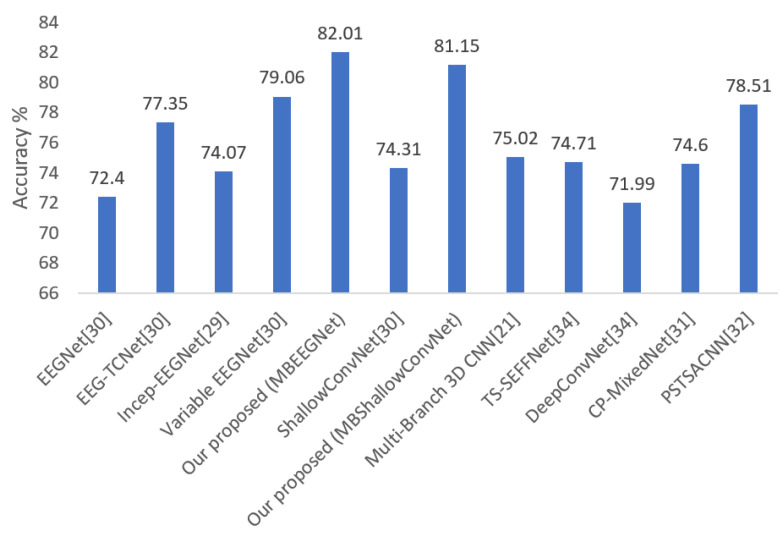
Average classification accuracy on the MI BCI IV-2a dataset.

The average classification accuracy of our proposed models (MBEEGNet and MBShallowConvNet) compared with the fixed EEGNet, EEG-TCNet, Incep-EEGNet, variable EEGNet, Multi-Branch 3D CNN [21], TS-SEFFNet [34], DeepConvNet [34], CP-MixedNet [31], and parallel spatial-temporal self-attention CNN (PSTSACNN) [32] are shown in Figure 7. The proposed models have an accuracy of at least 6.13% higher than the most competitive networks, as seen in the graph.

Furthermore, the accuracy of the proposed MBEEGNet is at least 2.95% higher than the variable network. This implies that the system’s performance was enhanced by features from separate branches with different kernel sizes.

Table 4 and Table 5 summarize the precision and recall for each class of the proposed models per subject, and, also, we provide the average of precision, recall, and F1 scores. Table 6 shows some results of the experiments performed to choose the best combination of hyperparameters and activation functions.

**Figure 8:**
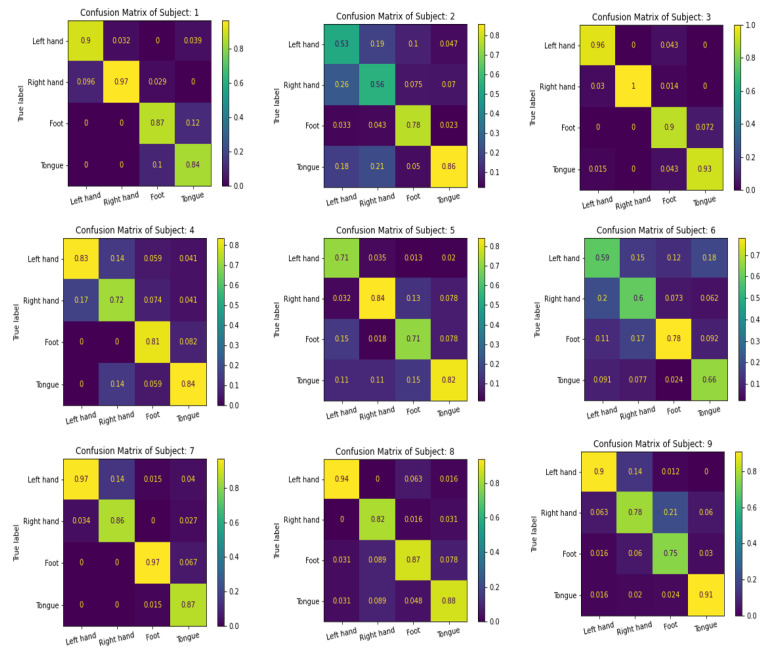
The confusion matrixes of MBEEGNet on the MI BCI IV-2a dataset.

**Figure 9:**
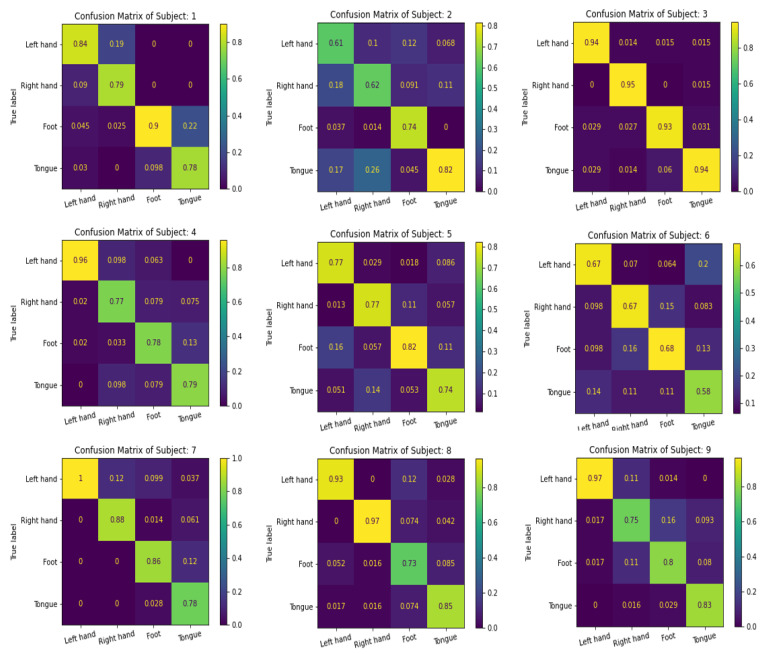
The confusion matrixes of MBShallowConvNet on the MI BCI IV-2a dataset.

We use the confusion matrix to test the efficiency of the feature extracted by the proposed multibranch models MBEEGNet and MBShallowConvNet, and the results for the nine subjects in the MI BCI IV-2a dataset are shown in Figure 8 and Figure 9.

We compute the number of parameters in each of our multibranch models, and compare it to other single and multiscale approaches to further analyze the computational complexity of the suggested networks. The findings are shown in Table 7. The proposed MBEEGNet and MBShallowConvNet have roughly 8.908 × 10^3^ and 147.22 × 10^3^ parameters, respectively, which are fewer than the other multiscale models, TS-SEFFNet and CP-MixedNet, which have 282 × 10^3^ and 836 × 10^3^ parameters. Moreover, the MBEEGNet has fewer parameters than the single-scale DeepConvNet and ShallowConvNet models.

The variable network has a problem with generalizing to each person; it is more subject-specific. The fixed network, which has fixed parameters for all subjects in the experiments, is more generalized for any practical application in general. Our proposed method has fixed parameters, which is more generalized for general applications, whereas the variable nets are subject-specific, so they do not work for all the common people. If we compare with the fixed EEGNet, which has 2.63 K parameters, our fixed proposed method (MBEEGNet) has 8.908 K parameters, but, with this expense, we achieved almost 10% higher accuracy than that. If we compare between variable EEGNet and our proposed method (MBEEGNet), the variable EEGNet has 15.6 K parameters, and ours has 8.908 K parameters, and we improve the accuracy by around 3%.

### 4.3. Results of HGD

Table 8 shows a summary of classification accuracy in the second dataset (HGD). From the table, we can see that the multibranch model achieves better accuracy than the single branch in both proposed models.

**Figure 10:**
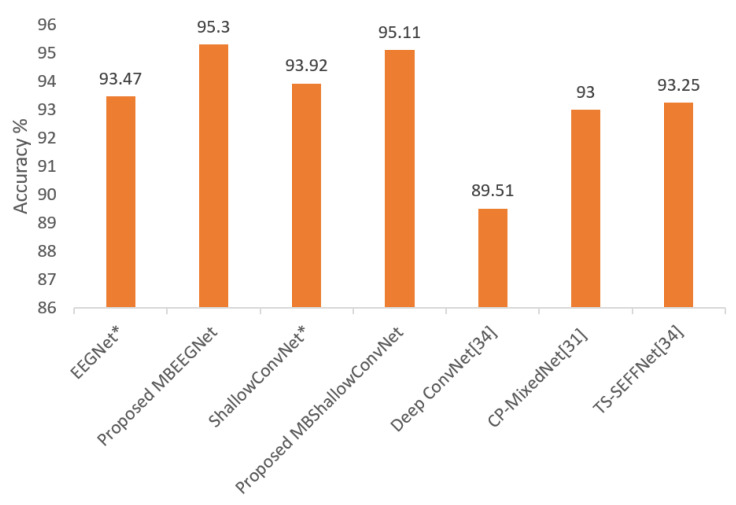
Average classification accuracy on the HGD; * means reproduced.

The average classification accuracies of our multibranch proposed models (MBEEGNet and MBShallowConvNet) compared with the single-scale models, EEGNet, ShallowConvNet, DeepConvNet, and other multiple scales networks, TS-SEFFNet [34] and CP-MixedNet [31], are shown in Figure 10.

Table 9 and Table 10 summarize the precision and recall for each class of the proposed models per subject, and, also, we provide the average of precision, recall, and F1 scores.

## 5. Conclusions

Multibranch models, which concatenated features from many branches of a basic model before classifying them using a softmax layer, were suggested. The models’ goal was to employ global parameters for all subjects that can outperform fixed hyperparameters in current models, while also remaining similar to or exceeding models that used variable hyperparameter networks for each subject. Our results achieved the goal, with higher accuracy than fixed and variable networks with less human intervention. The research was conducted using two publicly available datasets: the BCI Competition IV-2a dataset and the HGD dataset. The within-subject approach was used in the experiment, and global hyper-parameters were used for all subjects in both datasets. On the four-class MI set (BCI-IV 2a), the proposed MBEEGNet had an average classification accuracy of 82.01%, whereas the proposed MBShallowConvNet had an average classification accuracy of 81.15%. In MBEEGNet and MBShallowConvNet, the average accuracy on the HGD was 95.30% and 95.11%, respectively. We wish to keep enhancing BCI-MI classification models’ accuracy, and develop models that can be used in advanced BCI systems.

## Figures and Tables

**Table 1:** Summary of related work.

Related Work	Methods	Database	Acc%	Comment
Tang et al. [20]	5-layer CNN	Private, with two subjects and two classes	86.41% ± 0.77	It is one of the first papers that used a deep learning model to classify EEG-based MI. The method was tested on a private database.
Dose et al. [22]	Shallow CNN	Physionet EEG Motor Movement/MI Dataset	2classes 80.38% 3classes 69.8% 4classes 58.6%	As the number of classes increased, the accuracy dropped.
Sakhavi et al. [23]	FBCSP, C2CM	BCI competition IV-2a dataset	74.46% (0.659 kappa)	The authors used the DL model as a classifier only after they extracted features using a handcrafted approach.
Xu et al. [24]	Wavelet transform time-frequency images, two-layer CNN	Dataset III from BCI competition II and dataset 2a from BCI competition IV	92.75% 85.59%	This paper also used CNN as a classifier, and extracted the features from a combination of time-frequency images using wavelet transforms.
Zhao et al. [21]	Multi-branch 3D CNN	BCI competition IV-2a dataset	75.02% (0.644 kappa)	The 3D filter has more complexity, which makes it difficult to implement in real-time applications.
Amin et al. [25]	Multi-layer CNN-based fusion models: MLP +CNN (MCNN) autoencoder + CNN (CCNN)	BCI competition IV-2a dataset and HGD	75.7–95.4% 73.8–93.2%	Good accuracy using fixed parameters.
M. Riyad et al. [29]	Incep-EEGNet	BCI competition IV-2a	74.07%	They preprocessed the data (resample the signals at 128 Hz, and filter with a bandpass filter between 1 Hz and 32 Hz); also used cropping as data augmentation, and they trained the model with different learning rates in a large number of epochs.
T. M. Ingolfsson et al. [30]	EEG-TCNET	BCI competition IV-2a	77.35%	Good paper with good accuracy using fixed and variable parameters.
Y. Li et al. [31]	CP-MixedNet	BCI competition IV-2a dataset and HGD	74.6% 93.7%	It is a good model that has a multiscale in a part of it, but has a large number of parameters (836 K).
X. Liu et al. [32]	Parallel spatial-temporal self-attention CNN	BCI competition IV-2a dataset and HGD	78.51% 97.68%	A good paper that used self-attention in two parts.
Y. Li et al. [34]	TS-SEFFNet	BCI competition IV-2a dataset and HGD	74.71% 93.25%	It is a big model that has a large number of parameters (282 K).

**Table 2:** Global hyper-parameters used for all subjects in MBEEGNet.

Branch	Hyperparameter	Value
First branch	Kernel size	16
	Number of temporal filters	4
	Dropout rate	0
Second branch	Kernel size	32
	Number of temporal filters	8
	Dropout rate	0.1
Third branch	Kernel size	64
	Number of temporal filters	16
	Dropout rate	0.2

**Table 3:** Classification accuracy (%) and κ-scores on the MI BCI IV-2a dataset.

←Subject	EEGNet [30]		EEG-TCNet [30]		Incep-EEGNet [29]		Variable EEGNet [30]		Our Proposed MBEEGNet		ShallowConvNet [30]		Our Proposed MBShallow CovNet	
	Acc.	κ	Acc.	κ	Acc.	κ	Acc.	κ	Acc.	κ	Acc.	κ	Acc.	κ
S1	84.34	0.79	85.77	0.81	78.47	0.71	86.48	0.82	**89.59**	**0.86**	79.51	0.73	**82.58**	**0.77**
S2	54.06	0.39	65.02	0.53	52.78	0.37	61.84	0.49	**68.06**	**0.57**	56.25	0.42	**70.01**	**0.60**
S3	87.54	0.83	94.51	0.93	89.93	0.87	93.41	0.91	**94.58**	**0.93**	88.89	0.85	**93.79**	**0.92**
S4	63.59	0.51	64.91	0.53	66.67	0.56	73.25	0.64	**79.88**	**0.73**	80.90	0.75	**82.60**	**0.77**
S5	67.39	0.57	75.36	0.67	61.11	0.48	76.81	0.69	**76.92**	**0.69**	57.29	0.43	**77.81**	**0.70**
S6	54.88	0.39	61.40	0.49	60.42	0.47	59.07	0.45	**66.10**	**0.55**	53.28	0.38	**64.79**	**0.53**
S7	88.80	0.85	87.36	0.83	90.63	0.88	90.25	0.87	**91.57**	**0.89**	**91.67**	**0.89**	88.02	0.84
S8	76.75	0.69	83.76	0.78	82.29	0.76	87.45	0.83	**87.71**	**0.84**	81.25	0.75	**86.91**	**0.83**
S9	74.24	0.65	78.03	0.71	**84.38**	**0.79**	82.95	0.77	83.69	0.78	79.17	0.72	**83.83**	**0.78**
Mean	72.40	0.63	77.35	0.70	74.07	0.65	79.06	0.72	**82.01**	**0.76**	74.31	0.66	**81.15**	**0.75**
S. D.	13.27	0.18	11.57	0.15	14.06	0.19	12.28	0.16	**10.13**	**0.13**	14.54	0.19	**9.04**	**0.12**

In this table, the bold values indicate the best results, Acc. is the accuracy and S. D. is the standard deviation.

**Table 4:** Precision, recall, and F1 Score on the MI BCI IV-2a dataset using MBEEGNet.

		1	2	3	4	5	6	7	8	9	Average
**Precision**	LH	90.36	52.84	95.52	83.00	70.86	59.54	96.61	93.81	90.45	81.44
	RH	96.81	55.83	100	72.00	83.75	60.18	86.00	82.16	78.00	79.41
	F	87.09	77.61	90.00	80.84	70.79	78.23	97.00	87.26	75.30	82.68
	Tou.	84.08	86.00	92.81	83.67	82.33	66.40	86.65	87.56	91.00	84.50
	Avg.	89.58	68.07	94.58	79.88	76.93	66.09	91.57	87.70	83.69	82.01
**Recall**	LH	92.69	61.13	95.71	77.57	91.26	56.73	83.26	92.25	85.55	81.79
	RH	88.58	58.03	95.78	71.64	77.78	64.17	93.38	94.58	70.08	79.34
	F	87.88	88.74	92.59	90.81	74.27	67.71	93.54	81.46	87.62	84.96
	Tou.	89.36	66.15	94.13	80.85	68.91	77.46	98.30	83.97	93.81	83.66
	Avg.	89.63	68.51	94.55	80.22	78.05	66.52	92.12	88.06	84.27	82.44
**F1 Score**	Avg.	89.61	68.29	94.57	80.05	77.49	66.30	91.84	87.88	83.98	82.22

In this table, LH: left hand, RH: right hand, F: feet, Tou.: tongue.

**Table 5:** Precision, recall, and F1 Score on the MI BCI IV-2a dataset using MBShallowConvNet.

		1	2	3	4	5	6	7	8	9	AVG.
**Precision**	LH	83.58	61.18	94.19	96.00	77.46	66.60	100	93.09	96.61	85.41
	RH	78.61	62.37	94.53	77.08	77.31	66.34	88.00	96.81	76.06	79.68
	F	90.18	74.30	92.54	77.92	81.92	67.73	85.91	73.15	79.76	80.39
	Tou.	78.00	82.16	93.91	79.40	74.52	58.41	78.16	84.58	82.75	79.10
	Avg.	82.59	70.00	93.79	82.60	77.80	64.77	88.02	86.91	83.80	81.14
**Recall**	LH	81.55	67.93	95.53	85.64	85.27	66.73	79.62	86.27	88.66	81.91
	RH	89.77	61.94	98.45	81.57	81.05	66.93	92.15	89.32	73.53	81.63
	F	75.63	93.55	91.44	81.00	71.49	63.67	87.75	82.67	79.44	80.74
	Tou.	85.90	63.32	90.12	81.70	75.20	61.70	96.53	88.82	94.86	82.02
	Avg.	83.21	71.68	93.89	82.47	78.25	64.76	89.01	86.77	84.12	81.58
**F1 Score**	Avg.	82.90	70.83	93.84	82.54	78.03	64.76	88.51	86.84	83.96	81.36

In this table, LH: left hand, RH: right hand, F: feet, Tou.: tongue.

**Table 6:** Accuracy for proposed models at different parameter combinations.

Methods	Hyperparameters	Activation Function	Average Accuracy (%)
MBEEGNet	B1:F1 = 8, KE = 32, Pe = 0.2 B2:F1 = 16, KE = 64, Pe = 0.1 B3:F1 = 32, KE = 128, Pe = 0	Relu	77.03
	B1:F1 = 8, KE = 32, Pe = 0.2 B2:F1 = 16, KE = 64, Pe = 0.1 B3:F1 = 32, KE = 128, Pe = 0	elu	80.30
	B1:F1 = 4, KE = 16, Pe = 0 B2:F1 = 8, KE = 32, Pe = 0.1 B3:F1 = 16, KE = 64, Pe = 0.2	Relu	78.63
	B1:F1 = 4, KE = 16, Pe = 0 B2:F1 = 8, KE = 32, Pe = 0.1 B3:F1 = 16, KE = 64, Pe = 0.2	elu	82.01
MBShallowConvNet	KE1 = 10, KE2 = 20, KE3 = 30	-	80.36
	KE1 = 15, KE2 = 25, KE3 = 35	-	78.63
	KE1 = 5, KE2 = 15, KE3 = 20	-	81.15

In this table, B1, B2, B3 mean branch 1, 2, 3, respectively.

**Table 7:** Comparison of the number of parameters and mean accuracy.

Methods	Mean Accuracy (%)	Number of Parameters
DeepConvNet [32]	71.99	284 × 10^3^
EEGNet [29]	72.40	2.63 × 10^3^
ShallowConvNet [29]	74.31	47.31 × 10^3^
TS-SEFFNet [32]	74.71	282 × 10^3^
CP-MixedNet [32]	74.60	836 × 10^3^
EEG-TCNet [29]	77.35	4.27 × 10^3^
Variable EEGNet [29]	79.06	15.6 × 10^3^
Our proposed (MBEEGNet)	82.01	8.908 × 10^3^
Our proposed (MBShallowConvNet)	81.15	147.22 × 10^3^

**Table 8:** Classification accuracy (%) on the HGD.

*Methods/Subj.*	1	2	3	4	5	6	7	8	9	10	11	12	13	14	Mean	Std. Dev.
EEGNet *	94.37	92.50	100	96.25	96.87	98.12	93.07	96.87	98.12	91.25	80.00	96.25	95.60	79.37	93.47	6.30
*MBEEGNet*	95.02	95.02	100	99.40	98.17	98.80	93.13	95.52	98.18	92.14	89.43	96.02	94.45	88.88	95.30	3.50
ShallowConvNet *	96.87	93.75	99.37	98.12	98.12	93.12	92.45	96.87	98.12	90.62	76.25	95.00	94.96	91.25	93.92	5.79
*MBShallowConvNet*	98.25	96.23	98.80	98.18	97.65	96.90	93.80	97.00	97.52	92.50	80.78	96.25	95.62	92.04	95.11	4.62
*DeepConvNet [34]*	81.88	91.88	93.13	92.50	90.63	93.13	84.28	90.80	96.88	85.00	88.13	91.25	89.94	83.75	89.51	4.32
*TS-SEFFNet [34]*	90.69	93.53	98.53	96.88	92.90	93.53	92.40	91.78	96.88	89.88	92.78	95.40	93.03	87.34	93.25	2.97

* Reproduced.

**Table 9:** Precision, recall, F1 score, and κ-score on the HGD using MBEEGNet.

		1	2	3	4	5	6	7	8	9	10	11	12	13	14	AVG.
**Precision**	LH	92.81	95.19	100	100	95.19	97.61	92.54	97.10	97.61	95.00	79.56	100	92.09	84.34	94.22
	RH	94.81	97.49	100	100	100	100	90.27	85.00	95.10	93.00	88.56	100	88.09	83.08	93.96
	F	97.39	94.72	100	100	97.49	100	95.00	100	100	93.91	95.19	87.00	100	88.09	96.34
	Tou.	95.10	92.72	100	97.61	100	97.61	94.71	100	100	86.61	94.38	97.10	97.61	100	96.67
	Avg.	95.03	95.03	100	99.40	98.17	98.80	93.13	95.52	98.18	92.13	89.42	96.02	94.45	88.88	95.30
**Recall**	LH	97.28	97.44	100	100	100	100	92.72	86.61	100	95.38	93.71	100	90.64	81.47	95.38
	RH	92.77	97.59	100	100	97.66	97.66	94.74	96.70	97.54	100	81.65	100	91.76	84.35	95.17
	F	93.00	90.73	100	97.66	97.58	97.66	94.72	100	95.33	81.95	97.04	96.77	95.42	92.15	95.00
	Tou.	97.34	94.61	100	100	97.56	100	90.56	100	100	93.38	87.04	88.18	100	97.66	96.17
	Avg.	95.10	95.09	100	99.41	98.20	98.83	93.18	95.83	98.22	92.68	89.86	96.24	94.46	88.91	95.43
**F1 Score**	Avg.	95.06	95.06	100	99.41	98.18	98.82	93.16	95.68	98.20	92.40	89.64	96.13	94.45	88.89	95.36
**κ-** **Score**	Avg.	93.37	93.36	100	99.40	97.56	98.40	90.84	94.03	97.57	89.52	85.90	94.70	92.60	85.18	93.73

Where LH: left hand, RH: right hand, F: feet, Tou.: tongue.

**Table 10:** Precision, recall, F1 score, and κ-score on the HGD using MBShallowConvNet.

		1	2	3	4	5	6	7	8	9	10	11	12	13	14	AVG.
**Precision**	LH	100	97.61	100	97.61	93.09	97.39	86.00	97.29	95.19	92.81	73.15	97.39	97.39	91.91	94.06
	RH	93.00	97.39	100	97.49	100	100	94.28	90.73	97.49	92.72	65.59	97.61	92.54	83.75	93.04
	F	100	94.81	100	100	100	95	97.49	100	97.39	91.91	90.27	95.00	97.39	92.54	96.56
	Tou.	100	95.10	95.19	97.61	97.49	95.19	97.49	100	100	92.54	94.00	95.00	95.19	100	96.77
	Avg.	98.25	96.23	98.80	98.18	97.65	96.90	93.81	97.00	97.52	92.50	80.75	96.25	95.63	92.05	95.11
**Recall**	LH	97.75	100	100	100	100	95.10	93.78	91.25	100	97.48	72.35	95.19	95.10	86.55	94.61
	RH	100	95.10	100	97.59	97.75	97.47	87.04	97.12	97.59	95.09	75.23	100	92.45	88.79	94.37
	F	100	92.68	95.42	95.33	95.42	95.19	97.49	100	95.19	86.14	92.98	94.91	95.19	93	94.92
	Tou.	95.51	97.34	100	100	97.68	100	97.49	100	97.47	91.99	82.10	95.00	100	100	96.75
	Avg.	98.32	96.28	98.85	98.23	97.71	96.94	93.95	97.09	97.56	92.68	80.66	96.27	95.68	92.09	95.17
**F1 Score**	Avg.	98.28	96.25	98.83	98.20	97.68	96.92	93.88	97.05	97.54	92.59	80.71	96.26	95.66	92.07	95.14
**κ-** **Score**	Avg.	97.67	94.97	98.40	97.57	96.86	95.86	91.74	96.00	96.69	89.99	74.37	95.00	94.16	89.39	93.48

Where LH: left hand, RH: right hand, F: feet, Tou.: tongue.

## Data Availability

The BCI-IV-2a dataset can be downloaded from the following link: http://www.bbci.de/competition/iv/#dataset2a accessed on 30 December 2021, and the HGD dataset can be downloaded from the following link: https://gin.g-node.org/robintibor/high-gamma-dataset accessed on 30 December 2021.
